# Synthesis and Study of Novel Coumarin Derivatives Potentially Utilizable as Memory Media

**DOI:** 10.3390/molecules14124838

**Published:** 2009-11-25

**Authors:** Radoslav Flašík, Henrieta Stankovičová, Anton Gáplovský, Jana Donovalová

**Affiliations:** Institute of Chemistry, Faculty of Natural Sciences, Comenius University, Mlynská dolina CH-2, SK-842 15 Bratislava, Slovak Republic; E-Mails: rflasik@hotmail.com (R.F.); gaplovsky@fns.uniba.sk (A.G.); donovalova@fns.uniba.sk (J.D.)

**Keywords:** chromenes, photochromism, thermochromism

## Abstract

Novel coumarin derivatives, 2-oxo-2*H*-chromenecarbaldehyde hydrazones were prepared by reaction of substituted 2-oxo-2*H*-chromenecarbaldehydes with *N*-aminoimides in ethanol in the presence of 4-toluenesulfonic acid as catalyst. The photochromic and thermochromic properties of the prepared compounds were investigated.

## 1. Introduction

Information technology has revolutionized daily life and the continuosly increasing amount of data to be stored and manipulated has strongly stimulated the search for switching and memory elements as tiny as a single molecule. Molecular switches can be converted from one state to another by an external stimulus such as light, temperature, electricity or a chemical reaction. The operation of any optically controlled device is critically dependent upon the efficiency of light absorption by the input chromophore, and its ability to undergo electron transfer. Three characteristics make an excellent input chromophore: a large extinction coefficient, long-lived excited state, and a high fluorescence quantum yield. Photochromic compounds, where the reversible switching process is based on photochemically induced interconversions, are particularly attractive. Photochemical switching of mechanical devices [1], catalysts [2], transport systems [3], sensors [4] and surface properties of materials [5] are only a few applications that can be envisioned. Another important advantage of this type of systems is that light can be used for writing and processing information and, comparable to glass fiber technology, this would offer a highly increased speed compared to electronic addressing and switching.

Chromenes, especially 2-oxo-2*H*-chromenes (coumarins), have been extensively studied due to their commercial applications in several fields. These compounds have outstanding optical properties, including an extended spectral range, high quantum yield, superior photostability and good solubility in common solvents. Typical feature of chromene derivatives is that photophysical and spectroscopic properties can be readily modified by introduction of substituents in the chromene ring, giving themselves more flexibility to fit to various applications [6,7,8]. Coumarin derivatives are widely used as laser dyes [9], optical brighteners [10] and fluorescence markers [11].

The aim of this work was to synthesize new coumarin derivatives with C=N double bonds, which can potentially be used as a memory media. *E-Z* isomerization around C=N double bond can serve as a process on which a molecular switch is based [12]. The conversion from one isomer to the other can be initiated by light or temperature [13,14]. Besides *E-Z* isomerization around the C=N double bond, the possibility of conformer and tautomer formation should be considered, depending on the structure of adjacent fragments. Similar systems, 4-oxo-4*H*-chromene (chromone) derivatives with C=N bonds in position 3, were already synthesized [12].

## 2. Results and Discussion

### 2.1. Synthesis of 2-oxo-2H-chromenecarbaldehyde hydrazones

**Scheme 1:**
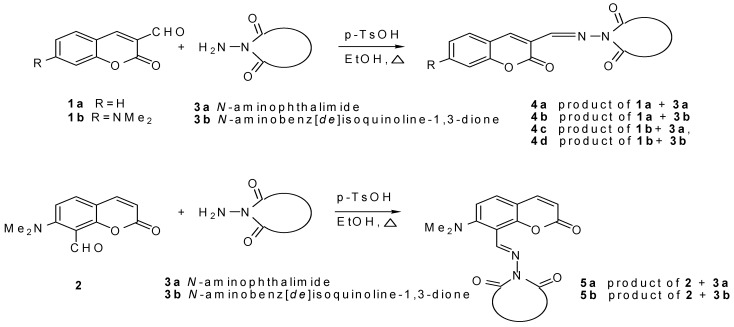
Synthesis of compounds **4** and **5**.

Reaction of substituted 2-oxo-2*H*-chromenecarbaldehydes **1** or **2** with *N*-aminoimides **3** afforded 2-oxo-2*H*-chromenecarbaldehyde hydrazones **4** or **5,** respectively (Scheme 1) under mild conditions.

The reaction of equimolar amounts of aldehyde **1** and imide **3** in alcohol in the presence of 4-toluenesulfonic acid as catalyst was carried out under reflux. The reaction undergoes quantitatively. Alcohols were found as the most suitable solvents for the synthesis. The isolation of products is very simple because they precipitate from a refluxing reaction mixture. Hydrazones **4** or **5** are stable crystalline products and can be easily purified by recrystallization, resp. trituration from alcohol.

### 2.2. Photochromism and thermochromism

**Figure 1:**
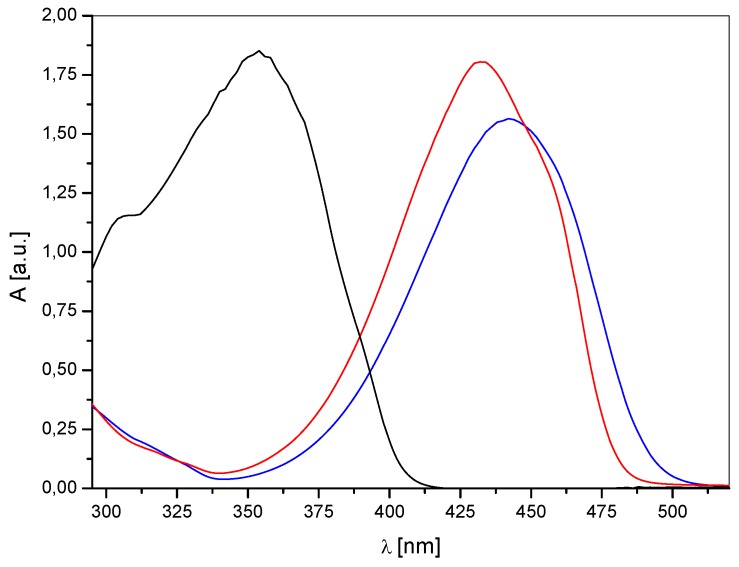
Absorption spectra of **4a** in methanol (**──**) and of **4c** in methanol (──), toluene (──).

2-Oxo-2*H*-chromene derivatives, depending on their structure, absorb light in a spectral range which is very suitable and attractive because readily available light sources can be used to store, resp. delete information. The photochromic (solvatochromic and fluorosolvatochromic) and thermochromic properties of synthesized compounds **4**, **5** were studied using UV-VIS and fluorescence spectroscopy. All studied compounds absorb light in the range λ = 200-520 nm. Their absorption maxima are bathochromically shifted (positive solvatochromism) few nanometers with increasing polarity of solvent (e.g. for **4c** λ_max-MeOH_ = 444 nm, λ_max-PhMe_ = 434 nm, Figure 1). This absorption corresponds to π→π∗ band. Introduction of electron donating substituent into position 7 of the 2-oxo-2*H*-chromene fragment induces a large shift of absorption maximum (e.g. for **4c** λ_max-MeOH_ = 444 nm, for **4a** λ_max-MeOH_ = 354 nm, Figure 1).

**Figure 2:**
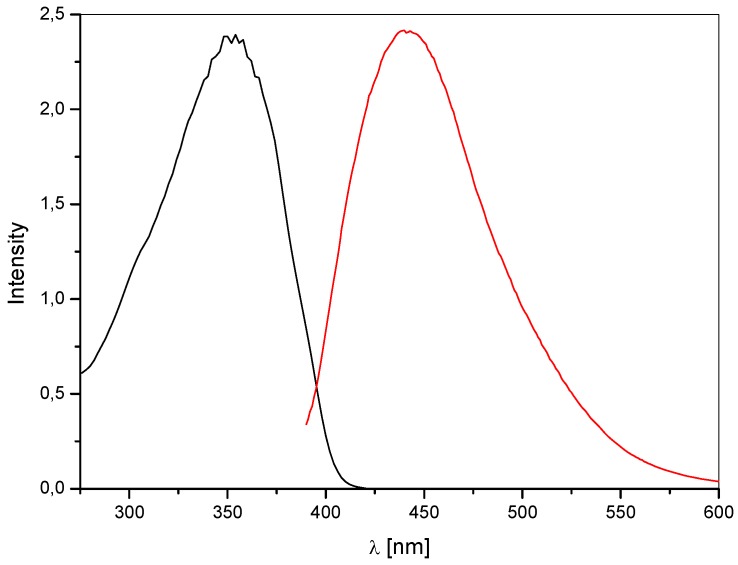
Absorption (**──**) and fluorescence emission (λ_ex_=360 nm ──) spectra of **4a** in methanol.

**Figure 3:**
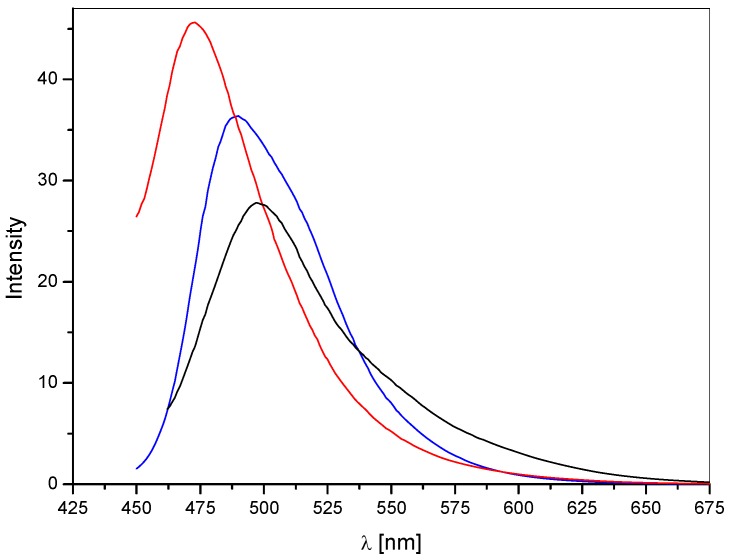
Fluorescence emission spectra of **4c** in toluene (λ_ex_= 490 nm ──) and of **4d** in toluene (λ_ex_= 430 nm ──) and in methanol ((λ_ex_= 430 nm ──).

**Figure 4:**
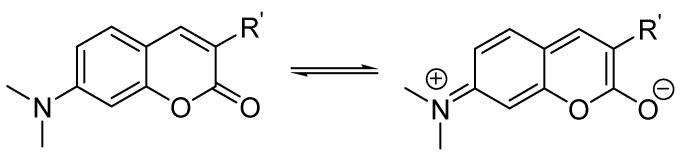
Bipolar structures.

Intensity of fluorescence emission of prepared compounds derived from unsubstituted aldehyde **1a** in methanol (Figure 2) is several-fold lower than for compounds derived from 7-*N,N*-dimethylamino-2-oxo-2H-chromene-3-carbaldehyde (Figure 3). 2-Oxo-2*H*-chromene-3-carbaldehyde hydrazones emit light with maximum of emission in the range of wavelenghts 420-460 nm. The maximum of the fluorescence emission is bathochromically shifted with increasing electron donating ability of substituent bound in position 7 of 2-oxo-2*H*-chromene fragment as well as with increasing solvent polarity. This shift confirms that the excited state of these compounds is more polar than their ground state. The dipole moment of the molecule increases after excitation, particularly of derivatives with dimethylamino substituent. Structure of molecule influences charge separation. Such “bipolar” structures (Figure 4) are better stabilized by more polar solvents.

**Figure 5:**
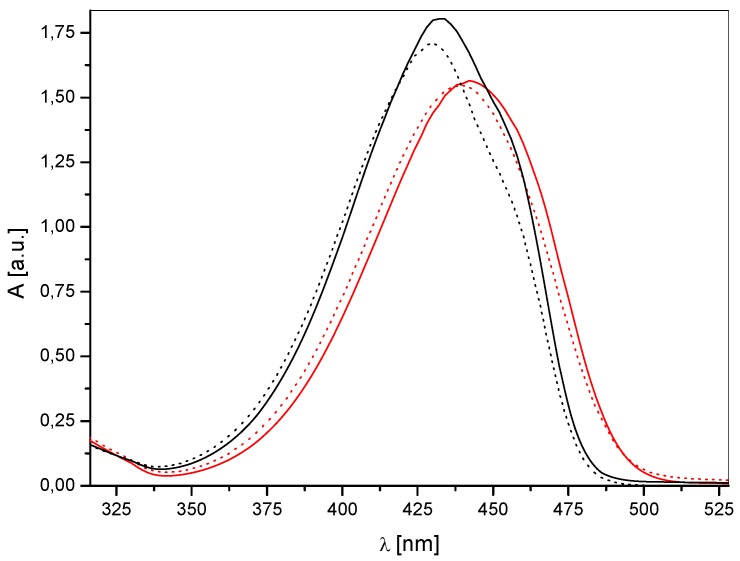
Absorption spectra of **4c** in methanol (*E*-isomer ──, *Z*-isomer ┄┄.) and in toluene (*E*-isomer **──**, *Z*-isomer **┄┄**.).

Irradiation of studied derivatives at λ ≅ 310 nm in methanol or toluene at room temperature induces hypsochromic shift of absorption maximum as well as decrease of intensity of long-wave absorption band. This change of long-wave band was assigned to *E-Z* isomerization around C=N bond (Figure 5).

**Figure 6:**
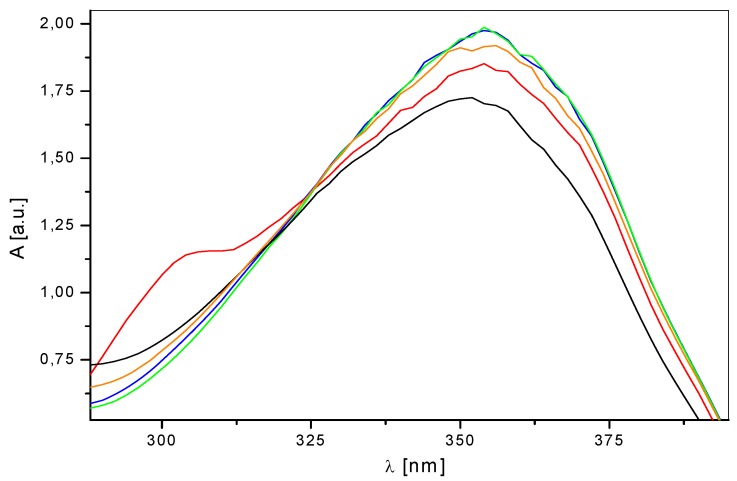
Absorption spectra of **4a** in methanol (dissolution of 4a (──), heating of solution to the boiling point of solvent (──), irradiation with visible light (halogen lamp, t=30 s, ──), irradiation with UV light (310 nm, t=15s, **──**), repeated heating of solution to the boiling point of solvent (──), repeated irradiation with UV light (310 nm, t=15s, ──).

**Figure 7:**
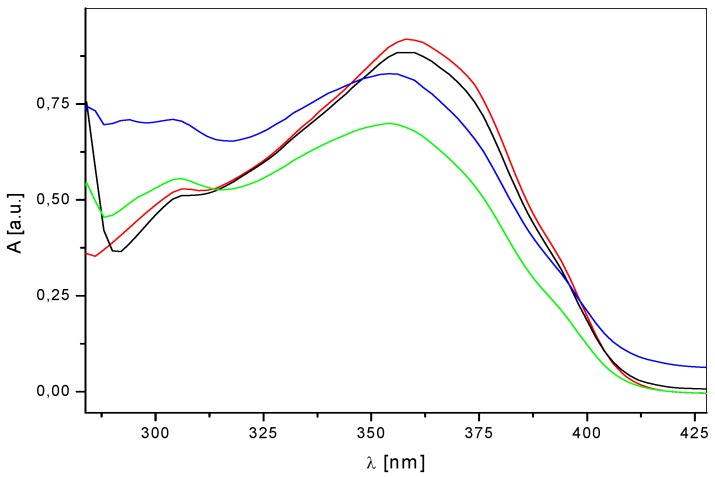
Absorption spectra of **4a** in toluene (dissolution of **4a** (──), heating of solution to the boiling point of solvent (**──**), irradiation with UV light (310 nm, t=15s, ──), repeated heating of solution to the boiling point of solvent (──).

Isomerization around the C=N bond is a reversible process. Retro-*Z-E* isomerization occurs photochemically with visible light or thermally in polar solvents (Figure 6). Thermal back isomerization takes place in non-polar solvents (Figure 7).

**Figure 8:**
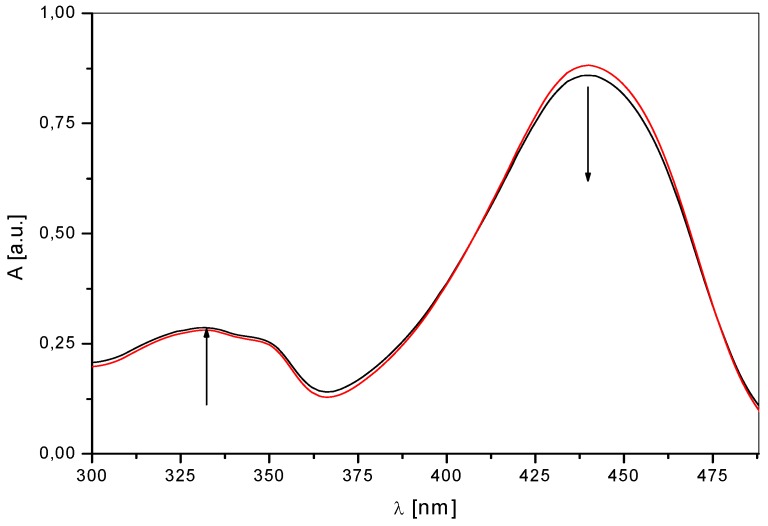
Absorption spectra of **4d** in methanol (sample irradiated after heating (──), sample heated after irradiation (**──**)).

When the compounds are dissolved, an increase of absorbance of the long-wave band is observed after standing a few minutes at room temperature. This change is probably due to reaching equilibrium between isomers, eventually conformers. This equilibrium is reached faster in polar than in non-polar media. Heating of solutions also causes fast reaching of equilibria between geometrical isomers. Irradiation of samples with UV-light induces hypsochromic shift of absorption maximum (the less stable isomer is formed in solution). Irradiation of this mixture with visible light causes bathochromic shift of absorption maximum. This process was repeated several times. The evidence for photochromic and thermochromic behaviour of studied compounds is, for examples of compound **4d**, also decrease of intensity of long-wave band (at λ = 450 nm) simultaneously with increase of intensity of absorption band at λ= 330 nm at irradiation of sample with UV-light followed by heating (Figure 8).

## 3. Experimental

### 3.1. General

Melting points (uncorrected) were measured on a Kofler hot stage. The ^1^H-NMR and ^13^C-NMR spectra were recorded at 300 MHz and at 75 MHz, respectively, on a Varian Gemini 200 spectrometer in CDCl_3_ or DMSO-d_6_ with tetramethylsilane as internal standard. Elemental analyses were performed on a Carlo Erba Strumentacione 1106 apparatus. Chemicals and solvents were purchased from the major chemical suppliers as highest purity grade. All solvents were dried by standard methods and distilled prior to use. Reactions with moisture-sensitive chemicals were performed under argon in an oven-dried flask. *N*-Aminobenz[*de*]isoquinoline-1,3-dione (**3b**) [15] and 2-oxo-2*H*-chromene-3-carbaldehyde (**1**) [16] were prepared according to literature procedures. For column chromatography Merck silica gel 60, 230-400 mesh was used with the indicated eluents.

### 3.2. General procedure for the preparation of 7-N,N-dimethylamino-2-oxo-2H-chromenecarbadehydes ***1b*** and ***2***

*Step 1- 4-N,N-dimethylamino-2-hydroxybenzaldehyde:* A solution of 3-*N,N*-dimethylaminophenol (21 g, 0.15 mol) in dry DMF (25 mL) was added dropwise to a Vilsmeier Haack adduct prepared from POCl_3_ (16.5 mL, 0.18 mol) and dry DMF (34 mL, 0.44 mol) at room temperature. The reaction mixture was stirred at room temperature for 15 minutes, at 37 °C for 15 minutes and at 85-90 °C for 30 minutes. After cooling, the reaction mixture was poured into crushed ice, the solution was neutralized with Na_2_CO_3_, the precipitate was filtered off, washed with water and dried. Mp 80.5-81 °C; 17.7 g, 70%; ^1^H-NMR (CDCl_3_): δ 3.08 (6H, s, CH_3_), 6.09 (1H, d, *J* = 2.1 Hz, H-3), 6.29 (1H, dd, *J* = 9, 2.1 Hz, H-5), 7.29 (1H, d, *J* = 9 Hz, H-6), 9.53 (1H, s, CHO), 11.6 (1H, s, OH).

*Step 2- 7-N,N-dimethylamino-2-oxo-2H-chromene:* A mixture of 4-*N,N*-dimethylamino-2-hydroxy-benzaldehyde (10 g, 60.5 mmol) and ethyl (triphenylphosphoranylidene)acetate (24.3 g, 69.8 mmol) was heated at 180 °C under argon for 1 hour. Method A [17]: after cooling, the crude product was purified by column chromatography on silica gel with hexane/ethyl acetate (2:1). Mp 163-165 °C; 11.7 g, 77%. Method B: after cooling, the crude product was crystallized from ethanol. Yield 6.93 g, 61%; ^1^H-NMR (CDCl_3_): δ 3.06 (6H, s, CH_3_), 6.07 (1H, d, *J* = 9.3 Hz, H-3), 6.5 (1H, d, *J* = 2.4 Hz, H-8), 6.59 (1H, dd, *J* = 8.7, 2.4 Hz, H-6), 7.27 (1H, d, *J* = 8.7 Hz, H-5), 7.57 (1H, d, *J* = 9.3 Hz, H-4).

*Step 3- 7-N,N-dimethylamino-2-oxo-2H-chromene-3-carbadehyde *(**1b**)*:* Method A: A solution of 7-*N,N*-dimethylamino-2-oxo-2*H*-chromene (1.5 g, 7.93 mmol) in dry DMF (40 mL) was added dropwise to a Vilsmeier Haack adduct prepared from POCl_3_ (3.7 mL, 40.3 mmol) and dry DMF (8 mL, 0.1 mol) at room temperature. The reaction mixture was stirred at 50 °C for 3 hours. After cooling, the reaction mixture was stirred at room temperature overnight, poured into crushed ice, the precipitate was filtered off, washed with water and dried. Mp 204-205 °C; 0.95 g, 55%; ^1^H-NMR (CDCl_3_): δ 3.12 (6H, s, CH_3_), 6.49 (1H, d, *J* = 2.4 Hz, H-8), 6.66 (1H, dd, *J* = 8.7, 2.4 Hz, H-6), 7.44 (1H, d, *J* = 8.7 Hz, H-5), 8.28 (1H, s, H-4), 10.14 (1H, s, CHO). Method B: A solution of 7-*N,N*-dimethylamino-2-oxo-2*H*-chromene (1.8 g, 9.51 mmol) in dry DMF (70 mL) was added dropwise to a Vilsmeier Haack adduct prepared from POCl_3_ (1.74 mL, 18.95 mmol) and dry DMF (1.46 mL, 18.95 mmol) at room temperature. The reaction mixture was stirred at 50 °C for 4 hours. After cooling, the reaction mixture was stirred at room temperature overnight, poured into crushed ice, the precipitate was filtered off, washed with water and dried. Yield 0.86 g of **1b**, 42%. The water from filtrate was partially evaporated, the concentrated filtrate was left to stand, after few days crystals of *7-N,N-dimethylamino-2-oxo-2H-chromene-8-carba*dehyde (**2**) were formed. The aldehyde **2** was filtered off and dried. Mp 223-225 °C; 0.6 g, 29%;^1^H-NMR (CDCl_3_): δ 3.04 (6H, s, CH_3_), 6.18 (1H, d, *J* = 9.4 Hz, H-3), 6.85 (1H, d, *J* = 8.9 Hz, H-6), 7.41 (1H, d, *J* = 8.9 Hz, H-5), 7.59 (1H, d, *J* = 9.4 Hz, H-4), 10.62 (1H, s, CHO); ^13^C-NMR (CDCl_3_): δ 44.21 (CH_3_), 108.89 (C-6), 110.09 (C-4a), 110.92 (C-3), 112.54 (C-8), 132.46 (C-5), 143.64 (C-4), 154.51 (C-4b), 159.61 (C-7), 160.33 (C-2), 186.07 (CHO); Anal. Calcd. for C_12_H_11_NO_2_ (217.2): C, 66.36; H, 5.1; N, 6.47. Found: C, 66.51; H, 4.94; N 6.45.

### 3.3. General procedure for the preparation of 2-oxo-2H-chromene-3-carbadehyde hydrazones ***4***

Aldehyde **1** was dissolved in 10 mL of hot absolute ethanol. *N*-Aminoimide **3** was added to the solution and after its dissolution a crystal of 4-toluenesulfonic acid was added to the reaction mixture. The mixture was refluxed for 15 minutes. After cooling, the precipitate was filtered off, washed with ethanol, dried and recrystallized from ethanol.

*3-[(N-phthalimidoyl)iminomethyl]-2-oxo-2H-chromene* (**4a**): Obtained from **1a** (50 mg, 0.29 mmol) and **3a** (47 mg, 0.29 mmol); Mp 265-267 °C; 67 mg, 73%; ^1^H-NMR (CDCl_3_): δ 7.33-7.43 (2H, m, Ar-H), 7.56-7.8 (6H, m, Ar-H), 8.33 (1H, s, H-4), 8.79 (1H, s, CH=); ^13^C-NMR (DMSO-d_6_): δ 116.21, 118.65, 120.59, 123.51, 124.95, 129.86, 130.15, 133.42, 134.99, 139.95, 150.75, 153.79, 159.53, 164.39; UV (methanol) λ_max_ 353 nm; Anal. Calcd. for C_18_H_10_N_2_O_4_ (318.3): C, 67.93; H, 3.17; N, 8.80. Found: C, 67.73; H, 3.16; N 8.82.

*3-[N-(1,3-dioxobenz[de]isoquinolinyl)iminomethyl]-2-oxo-2H-chromene* (**4b**): Obtained from **1a** (82 mg, 0.47 mmol) and **3b** (100 mg, 0.47 mmol); Mp 309 °C; 92 mg, 53%; ^1^H-NMR (CDCl_3_): δ 7.34-7.42 (2H, m, Ar-H), 7.63-7. 68 (2H, m, Ar-H), 7.74-7.78 (2H, t, *J*= 7.8 Hz, Ar-H), 8.27-8.30 (2H, d, *J* = 8.2 Hz, Ar-H), 8.67-8.70 (2H, d, *J* = 7.2 Hz, Ar-H), 8.87 (1H s, H-4), 8.91 (1H, s, CH=); ^13^C-NMR (CDCl_3_): δ 117.18, 118.87, 125.36, 127.26, 127.35, 130, 131.77, 131.89, 132.24, 133.99, 134.69, 134.76, 134.78, 142.63, 142.65, 164.82, 172.31; UV (methanol) λ_max_ 351 nm; Anal. Calcd. for C_22_H_12_N_2_O_4_ (368.1): C, 71.74; H, 3.28; N, 7.61. Found: C, 71.52; H, 3.29; N 7.59.

*7-(N,N-dimethylamino)-3-[(N-phthalimidoyl)iminomethyl]-2-oxo-2H-chromene* (**4c**): Obtained from **1b** (50 mg, 0.23 mmol) and **3a** (37.3 mg, 0.23 mmol); Mp 326-328 °C; 45 mg, 54%; ^1^H-NMR (CDCl_3_): δ 3.11 (6H, s, CH_3_), 6.64-6.65 (1H, d, *J* = 2.3 Hz, H-8), 6.83 (1H, dd, *J* = 8.7, 2.3 Hz, H-6), 7.75 (1H, d, *J* = 8.7 Hz, H-5), 7.90-7.95 (4H, m, Ar-H), 8.59 (1H, s, H-4), 9.24 (1H, s, CH=); ^13^C- NMR (DMSO-d_6_): δ 40.76 (CH_3_), 96.87 (C-8), 108.13 (C-6), 110.2 (C-4a), 111.48 (C-3), 123.29 (C_phth_-3,6), 129.95 (C-5), 131.26 (C_phth_-1,2), 134.77 (C_phth_-4,5), 140.80 (C-4), 150.15 (C-7), 153.55 (C-4b), 156.78 (CH=), 160.44 (C-2), 164.45 (C=O); UV (methanol) λ_max_ 434 nm; Anal. Calcd. for C_20_H_15_N_3_O_4_ (368.3): C, 66.48; H, 4.18; N, 11.63. Found: C, 66.28; H, 4.17; N 11.60.

*7-(N,N-dimethylamino)-3-[N-(1,3-dioxobenz[de]isoquinolinyl)iminomethyl]-2-oxo-2H-chromene* (**4d**): Obtained from **1b** (100 mg, 0.46 mmol) and **3b** (98 mg, 0.46 mmol); Mp 320-323 °C; 80 mg, 42%; ^1^H- NMR (CDCl_3_): δ 3.14 (6H, s, CH_3_), 6.52 (1H, d, *J* = 2.4 Hz, H-8), 6.67 (1H, dd, *J* = 9.8, 2.4 Hz, H-6), 7.44 (1H, d, *J* = 9.8 Hz, H-5), 7.79 (2H, dt, *J* = 8.4, 7.2, 0.9 Hz, Ar-H), 8.26 (2H, dd, *J* = 8.4, 0.9 Hz, Ar-H), 8.67 (2H, dd, *J* = 7.2, 0.9 Hz, Ar-H), 8.76 (1H s, H-4), 8.78 (1H, s, CH=); ^13^C-NMR (CDCl_3_): δ 40.52, 97.75, 97.86, 110.22, 110.49, 127.26, 131.27, 131.77, 132.01, 132.42, 134.49, 134.71, 143.16, 145.78, 161.45, 166.41, 166.43, 172.81; UV (methanol) λ_max_ 438 nm; Anal. Calcd. for C_24_H_17_N_3_O_4_ (411.4): C, 70.07; H, 4.17; N, 10.21. Found: C, 69.85; H, 4.16; N 10.20.

### 3.4. General procedure for the preparation of 2-oxo-2H-chromene-8-carbadehyde hydrazones 5

Aldehyde **2** was dissolved in hot absolute ethanol (10 mL). *N*-Aminoimide **3** was added to the solution and after its dissolution a crystal of 4-toluenesulfonic acid was added to the reaction mixture. The mixture was refluxed for 15 minutes. After cooling, the solvent was partially evaporated, the precipitate was filtered off, washed with ethanol, dried and recrystallized from ethanol.

*7-(N,N-dimethylamino)-8-[(N-phthalimidoyl)iminomethyl]-2-oxo-2H-chromene* (**5a**): Obtained from **2** (50 mg, 0.23 mmol) and **3a** (37.3 mg, 0.23 mmol); Mp 193-195 °C; 56 mg, 67%; ^1^H-NMR (CDCl_3_): δ 3.03 (6H, s, CH_3_), 6.19 (1H, d, *J* = 9.4 Hz, H-3), 6.91 (1H, d, *J* = 8.8 Hz, H-6), 7.38 (1H, d, *J* = 8.8 Hz, H-5), 7.60 (1H, d, *J* = 9.4 Hz, H-4), 7.77-7.82 (2H, m, Ar_phth_-H_β_), 7.9-7.95 (2H, m, Ar_phth_-H_α_), 9.61 (1H, s, CH=); ^13^C-NMR (DMSO-d_6_): δ 43.95 (CH_3_), 107.18 (C-8), 110.19 (C-6), 110.61 (C-4a), 113.27 (C-3), 123.26 (C_phth_-3,6), 130.03 (C-5), 130.67 (C_phth_-1,2), 134.72 (C_phth_-4,5), 144.51 (C-4), 154.48 (C-4b), 154.73 (C-7), 156.39 (CH=), 159.73 (C-2), 164.44 (C=O); UV (methanol) λ_max_ 371 nm; Anal. Calcd. for C_20_H_15_N_3_O_4_ (368.3): C, 66.48; H, 4.18; N, 11.63. Found: C, 66,51; H, 4.17; N 11.63.

*7-(N,N-dimethylamino)-8-[N-(1,3-dioxobenz[de]isoquinolinyl)iminomethyl]-2-oxo-2H-chromene* (**5b**): Obtained from **2** (100 mg, 0.46 mmol) and **3b** (98 mg, 0.46 mmol); Mp 283-285 °C; 105 mg, 55%; ^1^H- NMR (CDCl_3_): δ 3.17 (6H, s, CH_3_), 6.17 (1H, d, *J* = 9.4 Hz, H-3), 6.94 (1H, d, *J* = 8.9 Hz, H-6), 7.42 (1H, d, *J* = 8.9 Hz, H-5), 7.62 (1H, d, *J* = 9.4 Hz, H-4), 7.78-7.82 (2H, m, Ar-H), 8.24-8.29 (2H, m, Ar-H), 8.64-8.66 (2H, m, Ar-H), 9.2 (1H, s, CH=); ^13^C-NMR (CDCl_3_): δ 44.71, 111.12, 111.25, 112.71, 113.38, 127.25, 131.09, 131.76, 131.86, 132.65, 134.48, 134.70, 143.81, 143.9, 161.17, 161.45, 167.01, 186.25; UV (methanol) λ_max_ 339 nm; Anal. Calcd. for C_24_H_17_N_3_O_4_ (411.4): C, 70.07; H, 4.17; N, 10.21. Found: C, 69.99; H, 4.15; N 10.21.

### 3.5. Photochromism and thermochromism

UV-VIS spectra were recorded on Hewlett-Packard ‘Diode Array 8254’ spectrometer. Fluorescence spectra were taken in metanol on a Hitachi F2000 spectrometer (S_1_, S_2_ = 10 nm). All measurements were performed in a 1-cm thick absorption cell, resp. fluorescence cell, at room temperature. Photolysis experiments were performed in a quartz spectrophotometric cell using the filtered irradiation (λ ≅ 310 nm) from an UVP, Inc. Chromato VUE transilluminator, Model TM-15 or using unfiltered light from an Osram halogen lamp (150 W) in degassed methanol or toluene under nitrogen at room temperature.

## 4. Conclusions

2-Oxo-2*H*-chromenecarbaldehydes **1** or **2** react with *N*-aminoimides **3** to give 2-oxo-2*H*-chromenecarbaldehyde hydrazones **4** or **5** under mild conditions. It was found that *E*-*Z* isomerisation around the C=N bond takes place with ultraviolet light. This isomerization is a reversibile process. The back-*Z*-*E* isomerization occurs photochemically with the visible light in polar and in non-polar solvents and thermally only in non-polar solvents.
